# Deep-UV nitride-on-silicon microdisk lasers

**DOI:** 10.1038/srep21650

**Published:** 2016-02-18

**Authors:** J. Sellés, C. Brimont, G. Cassabois, P. Valvin, T. Guillet, I. Roland, Y. Zeng, X. Checoury, P. Boucaud, M. Mexis, F. Semond, B. Gayral

**Affiliations:** 1 Laboratoire Charles Coulomb (L2C), UMR 5221 CNRS-Université de Montpellier, F-34095 Montpellier, France; 2 Institut d’Electronique Fondamentale, CNRS, Univ Paris-Sud, Université Paris-Saclay, Bâtiment 220, Rue André Ampère, F-91405 Orsay, France; 3 Centre de Recherche pour l’Hétéro-Epitaxie et ses Applications (CRHEA)-CNRS, Rue Bernard Gregory, F- 06560, Valbonne, France; 4 Université Grenoble Alpes, F-38000 Grenoble, France; 5 CEA, INAC-PHELIQS, “Nanophysique et semiconducteurs” group, F-38000 Grenoble, France

## Abstract

Deep ultra-violet semiconductor lasers have numerous applications for optical storage and biochemistry. Many strategies based on nitride heterostructures and adapted substrates have been investigated to develop efficient active layers in this spectral range, starting with AlGaN quantum wells on AlN substrates and more recently sapphire and SiC substrates. Here we report an efficient and simple solution relying on binary GaN/AlN quantum wells grown on a thin AlN buffer layer on a silicon substrate. This active region is embedded in microdisk photonic resonators of high quality factors and allows the demonstration of a deep ultra-violet microlaser operating at 275 nm at room temperature under optical pumping, with a spontaneous emission coupling factor *β* = (4 ± 2) 10^−4^. The ability of the active layer to be released from the silicon substrate and to be grown on silicon-on-insulator substrates opens the way to future developments of nitride nanophotonic platforms on silicon.

The III-nitride semiconductors have been shown to be the most suitable materials for blue and near ultra-violet (UV) light sources as well as white-light domestic lighting^1^,^2^,^3^,^4^. The interest for III-nitride materials is not limited to these spectral ranges and efficient shorter wavelength light sources in the deep ultra-violet (UV-C) could trigger new developments in various domains including high density optical storage, water treatment, biochemical detection. Consequently, numerous studies have been devoted to light emitting and laser devices operating in the UV-C spectral range below 280 nm^5^,^6^,^7^. Achieving lasing at such short wavelength requires high structural quality materials with a good internal quantum efficiency (IQE). The usual strategy found in the literature is thus to grow low threading dislocation density nitride heterostructures on sapphire, SiC or bulk AlN substrates using complex combinations of thick engineered buffer layers, multistacking and in some cases epitaxial lateral overgrowth^8^,^9^. Low temperature and room temperature optically-pumped UV-C lasers have been demonstrated with ridge waveguide architectures but at the price of a significant complexity and expensive substrates (see^10^ for a review).

Another strategy to realize deep-UV laser sources relies on the use of small resonators with high quality factors. The high quality factors reinforce the interaction with the gain material and can lead to low thresholds and enhanced spontaneous emission coupling efficiency into the cavity mode^11^. Small dielectric resonators however require a layer with a thickness in the range of the operating wavelength and a strong index contrast for light confinement, thus ruling out the use of thick buffer layers. The simplicity of their fabrication and the use of thin layers are decisive advantages when designing such microlasers. The direct growth of nitride semiconductors on a silicon substrate offers several key advantages in this perspective. The fabrication of nitride resonators like microdisks is straightforward and high quality factor resonators have been demonstrated in the infra-red, green, blue and UV-range^12^,^13^,^14^,^15^,^16^, as reviewed in ref.^17^. One major challenge is to obtain optical gain even at room temperature, a requirement that is not obvious to achieve when growing directly very thin III-nitride layers on a silicon substrate: the first demonstration on such a layer of a photonic crystal laser in the blue spectral range has been reported very recently^18^. Active layers in such structures on silicon are at first sight not well suited for efficient emission at room temperature as they are highly dislocated and close to a hetero-interface, and thus much more prone to non-radiative recombinations than active layers grown in much thicker structures. In order to reach room temperature lasing for III-N microlasers, the challenge is thus to be able to obtain a large gain in a thin layer that allows for high quality factor microcavity fabrication.

In this work, we demonstrate microdisk lasers on a silicon substrate operating in pulsed mode at room temperature in the UV-C spectral range. This is achieved by combining the direct growth, on a Si or a Si-on-insulator (SOI) substrate, of ultrathin GaN/AlN binary quantum wells as active layers and the use of microdisks as resonators with high quality factors in the UV-C range. We show that this particular active layer leads to a narrow optical emission with a strong oscillator strength, with enough carrier confinement to maintain a large internal quantum efficiency at room temperature. These results open novel perspectives for the development of compact UV-C lasers on a silicon platform.

## Results

**Figure 1:**
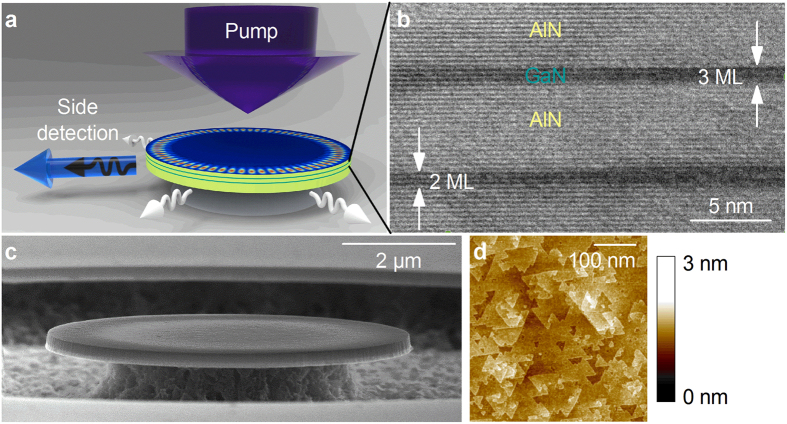
Structural properties of the nitride-on-silicon microdisks. (**a**) Schematic view of a microdisk. The structure is composed of an AlN disk with twenty GaN/AlN quantum wells (0.7 nm quantum well – 5 nm barrier), maintained by a silicon post on a silicon substrate; the distribution of the electric field intensity of a WGM is illustrated on the top of the disk, as calculated for a TE mode in a 2 μm-diameter microdisk (radial order *n *= 1, azimutal order *m *= 33, vertical confinement number *q *= 1). As indicated by arrows, the microdisk is excited at normal incidence by the pump laser and the emission is collected from the edge. (**b**) Transmission Electron Micrograph (TEM) of the GaN/AlN quantum wells. (**c**) Scanning Electron Micrograph (SEM) of a 8 μm-diameter microdisk. (**d**) Atomic Force Micrograph of the AlN buffer layer.

The microdisk structures are fabricated with a dedicated process for nitride nanophotonics (Fig. 1)^19^. The active layer is grown on a thin 100 nm AlN buffer layer onto a Si(111) substrate that can be selectively under-etched in order to define a microdisk or a free-membrane photonic crystal (Fig. 1.a). In order to reach emission in the deep-UV spectral range with a binary GaN/AlN heterostructure, ultra-thin GaN quantum wells (20 QWs) are grown, with a nominal thickness of only 2.8 monolayers (MLs). The transmission electron micrograph of the heterostructure (Fig. 1.b) reveals the sharp interfaces between the binary compounds GaN and AlN, and the interface fluctuations of the quantum wells. The quality of the QW interfaces is correlated to the small roughness (0.2 nm RMS) of the AlN buffer layer, as measured by atomic force microscopy (Fig. 1.d). The procedures of the NH_3_-assisted molecular beam epitaxy (MBE) growth and the microdisk fabrication are described in details in the Methods section. Such microdisks form optical resonators with a series of so-called whispering gallery modes (WGMs) circulating at the periphery of the disks (Fig. 1.a) and presenting a low modal volume and a high quality factor^20^. In the present samples, the electromagnetic field is vertically confined in a 200 nm thick multimode waveguide and the post diameter is 2.5 μm smaller than the disk diameter so to avoid WGM absorption from the post (Fig. 1.c)^14^.

**Figure 2:**
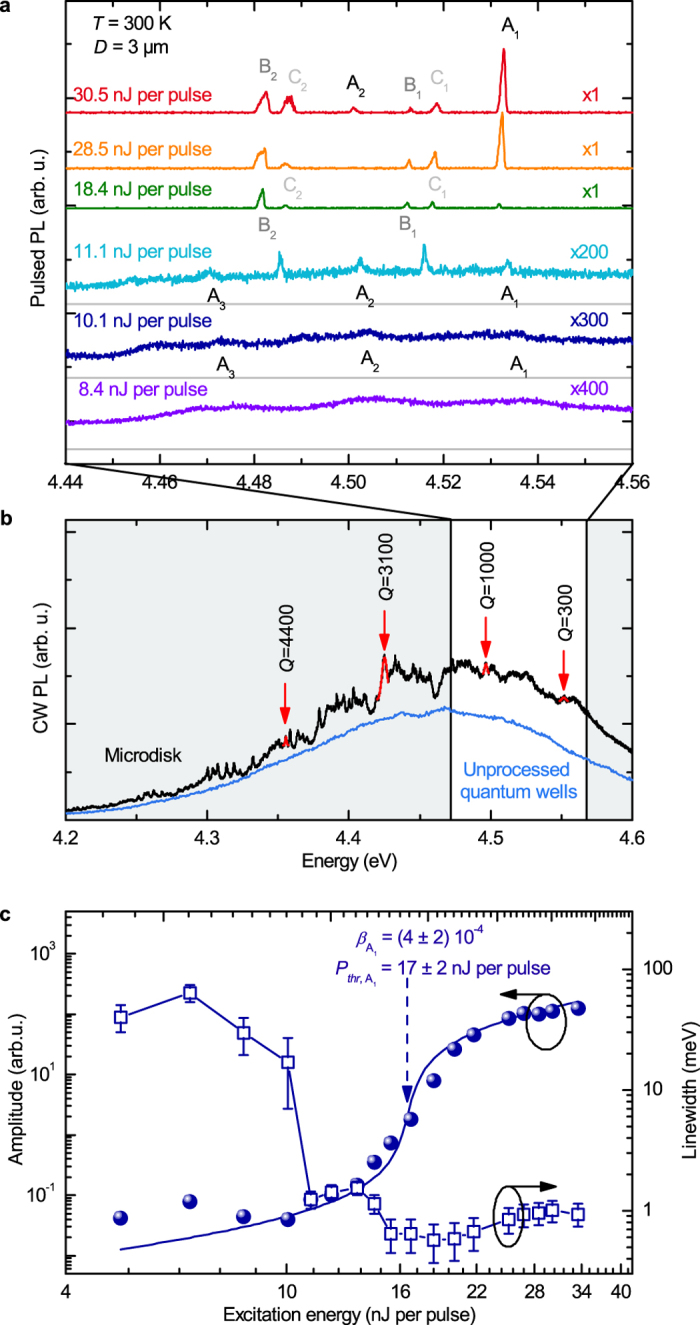
Room temperature lasing of a 3 μm-diameter microdisk. (**a**) Emission spectra under pulsed excitation taken at different pump powers (spectra are shifted for clarity). The spectral resolution is 0.17 meV. (**b**) PL spectrum of the same microdisk under continuous wave excitation, compared to the one of the un-processed active layer (excitation power density: 500 W.cm^−2^, same spectral resolution). The values of quality factors for some WGMs are indicated. (**c**) Integrated intensity (filled circles) and linewidth (empty squares) measured for lasing peak A_1_. The linewidth uncertainty is due to the slight irreversible energy shifts observed along the acquisitions at low excitation power, and carrier-induced or temperature-induced effects at high excitation power. The *β*-factor of the A_1_ mode is fitted by the rate equation model described in Supplementary Note 2 (plain blue line).

The lasing operation of a 3 μm-diameter microdisk is demonstrated in Fig. 2.a, under pulsed optical pumping at 266 nm and room temperature. A photoluminescence (PL) spectrum recorded in the linear regime under continuous (CW) excitation is shown in Fig. 2.b. The laser excites the microdisk through a microscope objective. The laser beam is intentionally defocused and the spot diameter (14 μm) is optimized for a proper excitation of the largest investigated microdisks. The emission is collected from the edge of the microdisks since the WGM propagate and leak out of the microdisk within the disk plane.

Under CW excitation (Fig. 2.b), the collected spectrum consists in the 200 meV-broad emission of the GaN/AlN QWs, with sharp peaks attributed to the WGMs; their eigennumbers cannot be unambiguously determined due to the multimode character of the waveguide, leading to a large number of observed modes. The WGM quality factor reaches *Q *= 4400 for the investigated 3 μm microdisk in the low energy tail of the spectrum, and decreases down to *Q *= 300 at high energy due to the onset of the QW absorption. Under low energy pulsed excitation (*P*<10 nJ per pulse) the WGMs appear as shallow structures in the collected spectrum. As the excitation pulse energy increases, sharp peaks appear, which intensity follows a strong nonlinear increase.

The input-output characteristics and the line narrowing has been studied into details for the peak A_1_ starting below threshold (Fig. 2.c); it is modeled by the standard rate equations for lasers (see Supplementary Note 2), leading to an emission coupling factor *β* = (4 ± 2) 10^-4^. The mode coupling and the quantum efficiency of the active layer are taken in account in our estimation of *β*. This value lies in the intermediate range between large modal volume lasers (*β* < 10^−7^) and the regime of single-mode microlasers operating with Purcell effect (*β* ~ 0.1–1)^18^. The *β*-factor is mainly limited by the multimode character of the investigated microdisks, that support 2 transverse-electric and 2 transverse-magnetic vertically confined modes, at least 2 families of radial modes, and 3 azimuthal modes efficiently coupled to the QW gain; smaller microdisks with reduced mode volume would be more appropriate for high-*β* microlasers. Finally the radiative efficiency of the active layer at room temperature is also expected to slightly degrade the *β*-factor. We observe slightly different thresholds for mode families A, B and C (differing through their radial and vertical eigennumbers). Those thresholds are also identified as steps in the power-dependence of the linewidth of the mode A_1_: it decreases from an absorption-limited value (26 ± 10 meV) at low power, to a regime of lower absorption (1.5 ± 0.5 meV) when only B_1_ and B_2_ modes are lasing (the active layer is almost transparent for the A_1_ mode), and a final low value (0.5 ± 0.2 meV) when the A_1_ mode enters lasing action. Due to the interplay between the multiple modes of the resonator, the input-output characteristics present evidences of strong mode competition beyond 2 *P*_*thr*_. An asymmetric lineshape is also observed for the lasing modes beyond 2 *P*_*thr*_, that is explained by the quasi-CW excitation scheme: the spectra are time-integrated over the 400 ps pulses, and slight energy shifts related to density-dependent effects or heating effects can occur within each pulse. The redshift of the modes observed as the pulse energy increases from below to above threshold is attributed to some photochemistry under UV irradiation that slightly modifies the dielectric properties of the disk as reported in ref.^21^. The onset of linewidth reduction below threshold is attributed to the bleaching of absorbing centers^22^.

**Figure 3:**
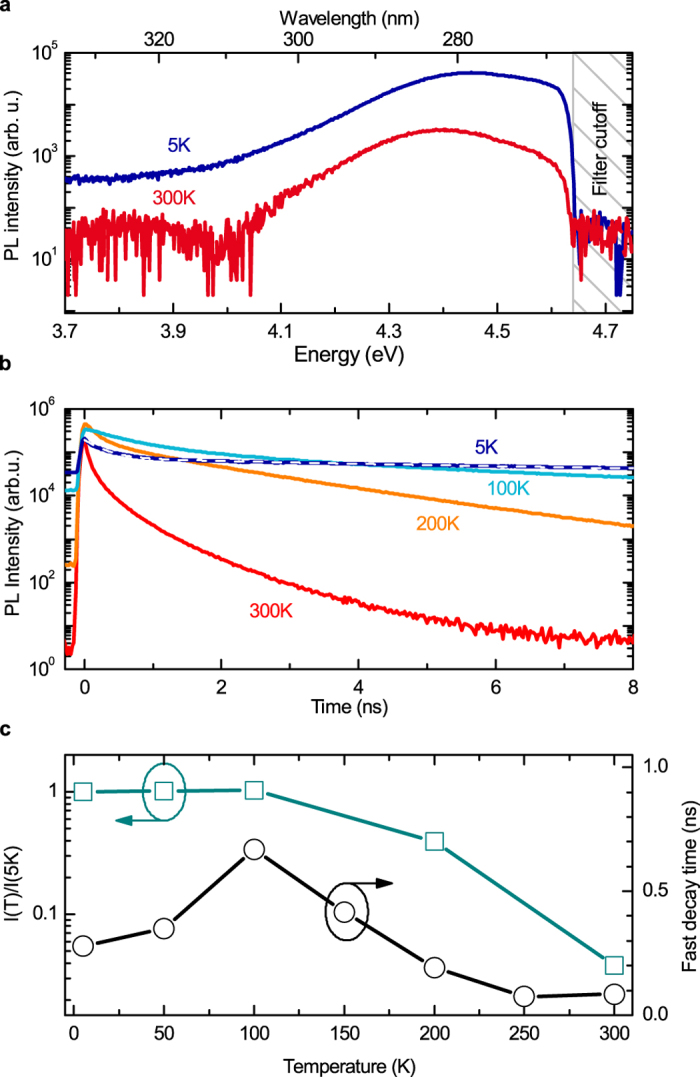
Optical properties of the active layer. (**a**) Photoluminescence of the active region at 5 K (full blue line) and 300 K (full red line). The QWs are excited by 200 fs pulses at 4.66 eV/266 nm. The grey dashed area represents the cutoff of the filter discriminating the photoluminescence from the scattered laser. (**b**) Time-resolved photoluminescence recorded at 5 K (blue line) and its bi-exponential decay fit (white dashed line). Experimental data at 100 K (cyan line), 200 K (orange line) and 300 K (red line) are also represented. (**c**) Integrated PL intensity normalized at *T *= 5 K (blue squares) and fast decay time (black dots) versus temperature.

The choice of an active layer for a deep-UV microlaser should rely on three main requirements. (i) The photonic geometries of microdisks and photonic crystal membranes that were successfully demonstrated in the UV range impose that the active layer forms a waveguide able to be released from the substrate; this excludes the use of thick buffer layers and short-period superlattices for strain relief, as well as the homoepitaxy on nitride substrates. (ii) The heterostructures can provide a practicable gain only if their emission is not too broad spectrally. (iii) This emission should be efficient at the operation temperature, i.e. the carriers should be strongly confined and their thermally-activated diffusion towards non-radiative centers should be low. The present GaN QWs are grown on a 100 nm-thick AlN buffer layer on Si, and thus satisfy the first above criterium. Their emission properties are presented on Fig. 3. The photoluminescence of the 2.8 MLs GaN QWs is peaked at 4.45 eV at low temperature and shifts to 4.40 eV at 300 K (Fig. 3.a). It should be emphasized that no signature of QW thickness fluctuations is observed: the transition associated to 4 ML areas of the QW, expected at 4.18 eV, may contribute to the low energy tail of the PL spectrum but no additional component related to 5 or 6 ML-QW is observed at lower energy. Together with the 200 meV full width at half maximum (FWHM) of the PL spectrum, this reflects a strong improvement of the QW spectral control compared to prior binary GaN/AlN quantum dots (QDs)^23^ or QWs^24^ (Supplementary Note 3), so that the present active layer compares well with the ternary AlGaN QWs developed for ridge lasers^9^. The inhomogeneous broadening of the transition is attributed to the in-plane exciton localization and to a possible inhomogeneous strain relaxation. The PL intensity decreases for temperatures larger than 100 K, where non-radiative processes start to be faster than radiative recombinations; it reduces by a factor 26 up to room temperature under 200 fs pulsed excitation and low power excitation density. The precise estimate of the internal quantum efficiency is discussed in details in the Supplementary Note 4, based on an extensive set of PL measurements. Assuming a saturation of non-radiative recombination channels under large excitation density at T = 5 K, the IQE could reach 10 to 40% under CW excitation, and even 80% under 400 ps pulsed excitation identical to the microdisk laser operation, which is a good and unexpected performance when considering the measured dislocation density of 7 10^10 ^cm^−2^. The room-temperature emission efficiency of the GaN/AlN QWs is therefore comparable to the one of AlGaN/AlGaN QWs grown on thick buffers on sapphire substrates^25^ despite the use of a very thin AlN buffer layer; it can obviously not compete with the most elaborate approaches of epitaxial lateral overgrowth and homoepitaxy on AlN substrates. The compatibility of the investigated GaN/AlN QWs with the three above criteria is a major breakthrough allowing the demonstration of the present deep UV microlaser.

In order to qualify the optical quality of GaN/AlN QWs, i.e. their potential to provide gain, we have measured their emission dynamics and its temperature dependence. This is even more important for binary GaN/AlN heterostructures grown along the c axis than for other materials due to the strength of the internal piezoelectric field^26^,^27^. The induced quantum-confined Stark effect (QCSE) separates the electron and hole wavefunctions. It drastically reduces the oscillator strength of the excitonic transition for heterostructures thicker than 5 MLs. This reduction reaches for example a factor 100 for QDs emitting around 400 nm^23^. The measurement of the PL decays is therefore performed under very low excitation power density (average excitation power 20 mW.cm^−2^), so to avoid any screening of the internal electric field, and it reveals a bi-exponential decay (Fig. 3.b). At low temperature, the fast component (300 ps) corresponds to the radiative recombination of the excitons confined in the QWs, thus assessing that the QCSE is negligible. Figure 3.c present the temperature dependence of the radiative lifetime and the QW intensity. The radiative lifetime increases by a factor 4 up to 100 K (Fig. 3.c), which could be explained as the temperature dependence of the radiative lifetime of excitons in 2D systems^28^,^29^,^30^ or as the redistribution of excitons within the disorder-induced density of states. The slow decay component is attributed to the carrier diffusion before recombination. Above 150 K the decrease of the fast slow decay time is a second signature of the onset of non-radiative recombinations.

**Figure 4:**
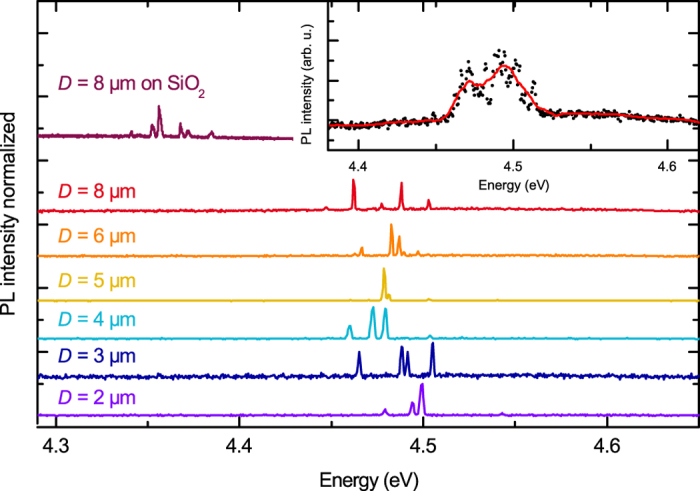
Microdisk laser emission versus disk diameter. Photoluminescence spectra taken above threshold for a wide range of microdisk diameters. Spectra are shifted for clarity. The top spectrum corresponds to a nitride microdisk directly lying on oxide following growth on a SOI substrate and underetching, and the other ones to nitride microdisks standing on a Si post. The spectrometer resolution is 1 meV. Inset: Average spectra (dots) of 20 investigated microdisks standing on a Si post (all diameters), recorded above lasing threshold at *P/P*_*thr *_= 1.3. The red line is a guide for the eye.

The robustness of the deep-UV microdisk laser is crucial for future prospects with other photonic designs. Figure 4 presents the spectra measured above threshold for a broad range of microdisk diameters (*D*), with a fixed diameter of the excitation spot of 14 μm. Contrary to previous works on bulk GaN^31^ and InGaN QWs microdisks^32^, lasing occurs for all investigated diameters and it is not limited to the microdisks smaller than 2 or 3 μm in diameter. Moreover the lasing threshold is rather constant for all microdisk diameters which actually indicates that the effective threshold power (if only the gain material spatially matched with the WGM were excited) decreases approximately linearly when the diameter decreases. There are very few studies on the threshold power vs diameter for microdisk lasers, the most complete one having been done on GaInAsP microdisks^33^ for which the authors show a dependence of the threshold current as the diameter squared, i.e. a constant threshold current density comparable to our observation.

Even if the gain of the active layer cannot be quantitatively measured in a microdisk laser, the gain spectrum is estimated by averaging over 20 microdisks brought into lasing conditions (Inset in figure 4): at a fixed ratio *P/P*_*thr *_= 1.3, the gain band stands around 4.48 eV, on the high energy side of the CW PL transition. Its spectral width is only 50 meV FWHM, a fraction of the PL inhomogeneous broadening. This can be understood as the frontier between the low energy part of the density of states, more sensitive to disorder, and higher energy states, that contribute the high spectral gain density of the present GaN/AlN QWs.

Finally the flexibility of the present nitride-on-silicon approach is demonstrated with a variant of the microlaser directly lying on oxide instead of forming a free-standing disk with a silicon post. This structure is first grown on a silicon-on-insulator (SOI) substrate, and the thin (~100 nm) Si layer is fully removed by under-etching of the microdisks. The oxide layer is only 200 nm thick. This microlaser embeds the same active layer, and lasing is also observed (Fig. 4 top) with a threshold typically twice larger than the one reported in Fig. 2.

To conclude, we demonstrate the first microlaser emitting in the deep UV range below 300 nm and at room temperature. It is based on the combination of a high quality microdisk resonator and a simple active layer based on thin binary GaN/AlN QWs grown on Si or SOI substrates. Such a design is much simpler, more compact and more versatile than the previously investigated active layers for lasers in the deep UV range and highlights all the advantages of growing nitrides on silicon. The emitters present a narrower emission than the other GaN/AlN heterostructures investigated to date, while reasonably preserving their radiative efficiency at room temperature. The combination of these three features opens the way to short wavelength integrated sources for UV photonics on silicon and SOI substrates.

## Methods

The sample is grown by NH_3_-MBE on Si(111) or SOI substrates. The structure consists of 100 nm AlN buffer layer grown at 1000°C followed by 20 pairs of GaN/AlN MQWs grown at 800°C. The barrier and the well thickness is 5 and 0.7 nm respectively. The microdisks are then fabricated by electron beam lithography using a positive photoresist (UV3) on a 150 nm thick silicon oxide hard mask. The oxide hard mask is etched by reactive ion etching while the nitride material is etched by chlorine-based inductively coupled plasma etching. The microdisks stand at the center of a 20 μm diameter area where the nitride material has been removed. The oxide hard mask is removed by buffered hydro-fluorhydric acid solution. The silicon is selectively underetched with XeF_2_. The underetching time controls the diameter of the microdisk post and the nitride is suspended in air on a ~1.25 μm radial distance. The size of the post is thus 2.5 μm smaller than the disk diameter. For microdisks on SOI substrates, the Si layer is fully removed and the nitride disk directly lies on SiO_2_.

Time-resolved photoluminescence measurements are performed with the third harmonics (266 nm, 200 fs pulses at 80 MHz) of a mode locked titanium sapphire laser. The signal is then analyzed in a 32 cm spectrometer and detected by a photomultiplier with a temporal resolution of 40 ps.

The linear and non-linear emission properties of the microdisks are investigated in a room-temperature micro-photoluminescence experiment. Two 266 nm lasers (CW and pulsed, with 400 ps long pulses, 50–4000 Hz repetition rate) are used in order to excite the microdisk through a microscope objective (Numerical aperture NA = 0.4). The emission is collected from the edge of the microdisks through a 7.5 cm lens and a multimode optical fiber (Collection NA = 0.1). It is analyzed in a 55 cm spectrometer and detected with a nitrogen-cooled CCD. The spectral resolution reaches 170 μeV at 4.4 eV with a 3600 lines per mm grating.

## Additional Information

**How to cite this article**: Sellés, J. *et al.* Deep-UV nitride-on-silicon microdisk lasers. *Sci. Rep.* **6**, 21650; doi: 10.1038/srep21650 (2016).

## Supplementary Material

Supplementary Information
