# Supplementary material for: Deep-UV nitride-on-silicon microdisk lasers
Source: Sci Rep. 2016 Feb 18;6:21650. doi: 10.1038/srep21650 (PMC4757875; doi:10.1038/srep21650)
Supplement: Supplementary Information [file srep21650-s1.pdf]

# Deep-UV nitride-on-silicon microdisk lasers

J. Sellés[1], C. Brimont[1], G. Cassabois[1], P. Valvin[1], T. Guillet[1,*] I. Roland[2], Y. Zeng[2], X. Checoury[2], P. Boucaud[2], M. Mexis[3], F. Semond[3], B. Gayral[4,5]

[1] *Laboratoire Charles Coulomb (L2C), UMR 5221 CNRS-Université de Montpellier, F-34095 Montpellier, France*

[2] *Institut d'Electronique Fondamentale (IEF), CNRS Université Paris Sud 11, F-91045, Orsay, France*

[3] *Centre de Recherche pour l'Hétéro-Epitaxie et ses Applications (CRHEA)-CNRS, Rue Bernard Gregory,F- 06560, Valbonne, France*

[4] *Université Grenoble Alpes, F-38000 Grenoble, France*

[5] *CEA, INAC-SP2M, « Nanophysique et semiconducteurs » group, F-38000 Grenoble, France*

*e-mail: thierry.guillet@umontpellier.fr

## Supplementary Note 1: Estimation of the microdisk laser threshold

The pump power density at the lasing threshold is a major figure in order to compare the present microlaser with other UV-C devices. Its estimate requires a lot of care since the microlaser is far from the textbook case of a ridge laser with a large area under a continuous and spatially homogeneous pump. Indeed the microdisks are pumped with 400 ps long pulses, a value comparable to the measured lifetime of the excitons in the QWs (see figure 3), so that the pumping regime is intermediate between ultrafast excitation and continuous excitation. The pump laser at 266 nm creates electron-hole pairs resonantly in the 20 QWs and its absorption can be roughly estimated to 10%, whereas it is more frequent to pump a QW laser above the energy of the barriers, and therefore benefit from a complete absorption of the pump. Moreover the area of the pump spot (14 µm diameter, 150 $\mu m^2$) is large compared to the area of the WGMs that are located only at the periphery of the disks, so that only a small fraction of the absorbed pump provides gain to the lasing modes. Finally the multimode character of the employed Q-switch pump laser leads to strong inhomogeneities and pulse-to-pulse fluctuations in the spot profile that cannot be accounted for in the estimation of the threshold. Assuming top-hat temporal and spatial profiles of the laser, the pump power density at threshold is estimated to 17 $MW.cm^{-2}$ for microdisks on silicon substrates and 40 $MW.cm^{-2}$ for microdisks lying on oxide layer. This value is one or two orders of magnitude larger than the best threshold densities demonstrated at 300K with an excitation above the barrier energy of a standard ridge laser[1–3] or of bulk-GaN and InGaN-QW based microdisk lasers [4,5]. It is however comparable to the values reported under a resonant excitation of the QW active layer[6]. Note that only 10% of the incident pump density is absorbed in the QWs and the effective pump power density threshold is in the $MW.cm^{-2}$ range.

## Supplementary Note 2: Estimation of the β-factor

In order to estimate the global emission coupling factor $\beta$, we use the theoretical model developed in the textbook "Optoelectronics" by E. Rosencher and B. Vinter[7]. Solving the rate equations for the carrier density $n$ and the photon density $s$ in a monomode cavity in the stationary regime allows relating them to the pump power density $P$:

$$\frac{P}{P_{tr}} = x + \frac{\beta}{\Gamma} \frac{x(x-1)}{\alpha - x}$$

$$s = A \frac{x}{\alpha - x}$$

where $P_{tr}$ the transparency power density, $x = \frac{n}{n_{tr.}}$ with $n_{tr}$ the transparency carrier density, $\alpha = \frac{n_{thr}}{n_{tr}}$ is the ratio between the carrier densities at threshold and at transparency, and $\Gamma$ is the spatial overlap between carriers and the photonic mode.

$\beta$ is here the product of the spontaneous emission coupling factor, and the quantum efficiency of the active medium ($\eta$).

Under the monomode approximation of the model, the prefactor of $s$ is equal to

$A = n_{tr}\, \beta\, (\alpha - 1) \frac{\gamma_{spon}}{\gamma_{cav}}$ , where $\gamma_{spon}$ is the quantum well radiative decay rate and $\gamma_{cav}$ is the cavity loss rate.

Inverting these two equations provides the power-dependence $s(P)$ of the photon density, that is compared to the experimental input-output characteristics of the microlaser, thus determining the microlaser parameters.

As shown in the Supplementary Note 4, the precise estimation of the quantum efficiency of the active layer is an intricate issue, so that we prefer to provide the global $\beta$-factor of the investigated microlaser. Moreover, the precise eigennumbers of the lasing modes being unknown, we assume for simplicity an overlap coefficient $\Gamma = 1$. For the $A_1$ mode presented in figure 2, the fitted $\beta$-factor ($\beta = (4 \pm 2).10^{-4}$) and the threshold pump power density ($P_{thr}$=17±2 nJ per pulse) are calculated for a value $\alpha = \frac{n_{thr}}{n_{tr}} = 1.7$, and a pump power density at transparency $P_{tr}$=10 nJ per pulse. Those values are compatible with the observation of the mode narrowing for this mode at $P=P_{tr}$.

**Supplementary Note 3: Influence of the quantum confined Stark effect and comparison between microdisks embedding GaN/AlN quantum dots and quantum wells**

Due to the strong internal electric field existing in GaN/AlN heterostructures, and the induced Quantum Confined Stark Effect (QCSE), the overlap between electrons and holes wavefunctions can be drastically reduced for QDs/QWs thicker than 5MLs that emit below 3.9 eV[8]. This results in a strong decrease of the oscillator strength of the optical transition.

Figure S1 presents the power dependence of PL emission for both thick QD (8—12 MLs) and thin QW (2.8 MLs) microdisks under CW and pulsed excitation. The microdisks have 2 and 4 µm diameter for CW measurements and have similar diameters (3 µm) for pulsed experiments. Their quality factors measured under CW excitation are rather similar (Q=4000 and 6000 for 2 and 4 µm respectively). For thick Stranski-Krastanov (SK) QDs, a large blueshift of about 700 meV is observed between low and high CW optical injection. This shift is a clear signature of the screening of the internal electric field under high excitation[9]. It should be emphasized that on the high energy tail (3.3-3.6 eV) the WGM intensity increases non-linearly but the broad emission underlying the peaks does the same; this cannot be attributed to any stimulated emission in the resonator[10]. The situation for thin QWs is different: no blueshift is observed even for high optical pumping. The QWs are thin enough so that the quantum confinement is stronger than the Stark effect and does not impede their oscillator strength.

Under pulsed excitation, lasing operation is obtained for the QW microdisk as already shown on figure 2 for a similar microdisk. In the case of the QD microdisk, we observe instead that the blueshift observed under CW excitation still increases. On the high energy tail (3.8-4 eV) of the QD microdisk emission, the contrast of the last visible WGMs slightly improves with excitation power. This can be attributed to the transition from absorption to transparency in this spectral range at large carrier densities. However the competition between the absorption of the QDs emitting at low energy and the nascent gain at high energy prevents the QD microdisks from lasing. The improved control of the emission linewidth of the QW active layer is therefore crucial in order to concentrate the gain on a narrower spectral range and to limit the detrimental absorption from thicker heterostructures as in the case of QD microdisks. We should emphasize that this situation is different to the one of InGaAs/GaAs microlasers and other III/V materials[11,12] for two reasons: (i) the control of the size distribution of InGaAs/GaAs QDs is much better than in our case. (ii) More importantly the QD thickness has a strong impact on their optical properties, since it induces a very strong variation of their emission energy (a large broadening), as well as a strong reduction of the oscillator

strength of their ground excitonic states (thick QDs are slow emitters from their ground states and strong absorbers at higher energy[9]). This last effect was not detrimental to laser action in InGaN/GaN QD microdisks[13] due to the low indium content of the QDs and the small induced electric field.

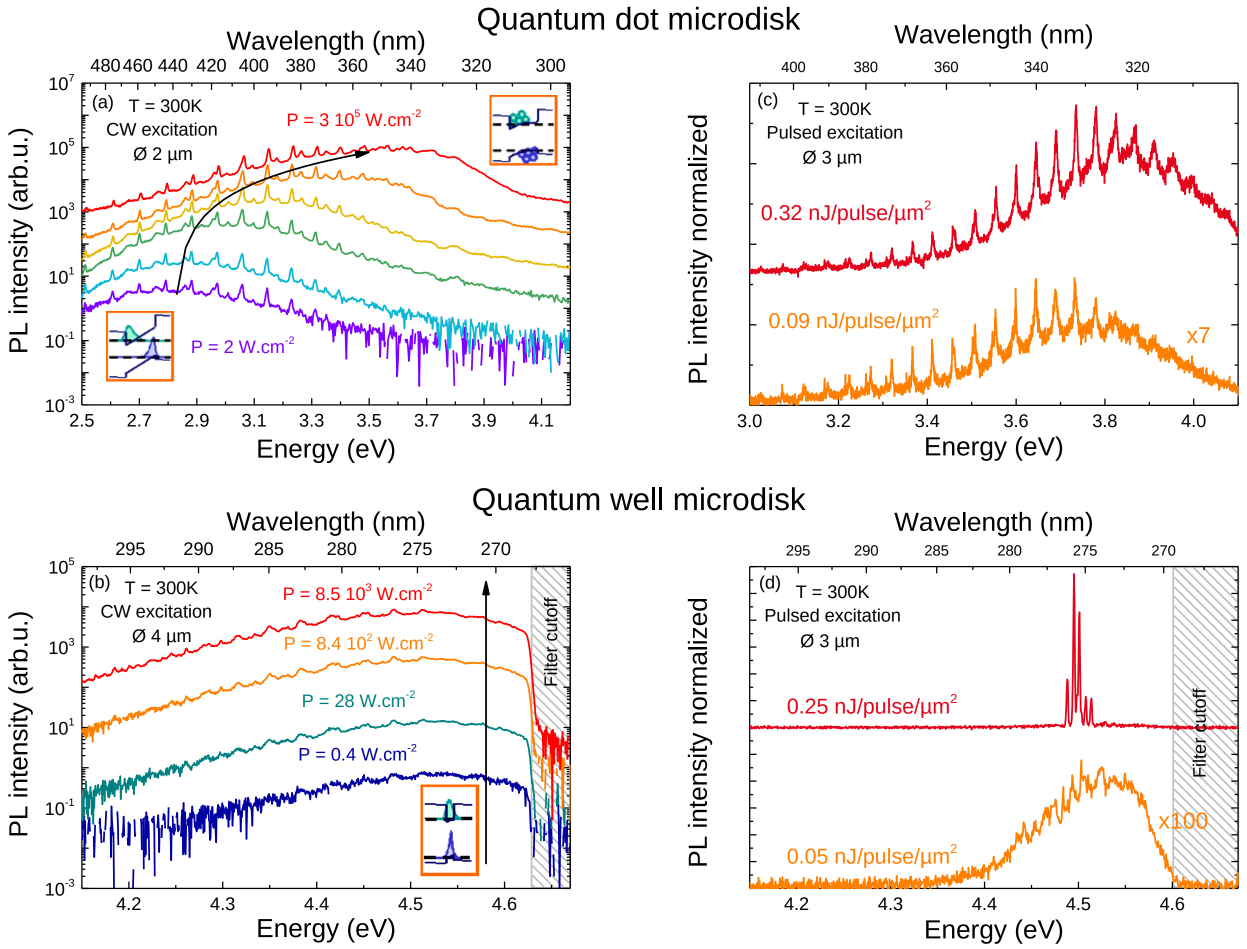


**Figure S1: Comparison of a QD microdisk and a QW microdisk under continuous wave and pulsed excitation**.

(a) Power-dependent photoluminescence spectra of a 2µm microdisk embedding 4 planes of GaN/AlN SK QDs. (b) Power-dependent photoluminescence spectra of a 4µm microdisk embedding 20 GaN/AlN QWs. (c) Photoluminescence spectra under pulsed excitation of a 3µm microdisk embedding 4 planes of GaN/AlN SK QDs. (d) Photoluminescence spectra under pulsed excitation of a 3µm microdisk embedding 20 GaN/AlN QWs. Both continuous wave and pulsed experiments are done at 4.66 eV/266 nm.

## Supplementary Note 4: Internal quantum efficiency of the GaN/AlN quantum wells

The estimation of the internal quantum efficiency (IQE) through photoluminescence experiments is an intricate issue. Figure S2.a presents the temperature-dependent PL spectra of a single GaN/AlN QW equivalent to the ones of the microdisk active layer under CW excitation at 5.07 eV (244nm). The spectra consist in a single peak which energy roughly follows the shift predicted from the Varshni law of the AlN barrier. As shown in Figure S2.b., the latter is close to the QW emission. The IQE is usually obtained from the ratio $I(300K)/I(5K)$ of the PL intensities at T=300K and 5K, assuming that the nonradiative processes are negligible at T=5K. However the IQE is defined as the number of emitted photons to the number per photo-created electron-hole pair, and it should better be calculated as the ratio of the intensity of the emission and the excitation power density (assuming a constant absorption coefficient at the laser energy vs power and temperature). Exploiting the ratio $I(300K)/I(5K)$ as an estimate of the IQE is therefore valid if the collected signal is proportional to the excitation power, but this assumption is not verified in our measurements. We prefer here to present the collected signal normalized to the excitation power density, $I(T)/P_{laser}$, as a function of temperature (figure S2.c). At T=5K the normalized signal increases by a factor 2 as the excitation increases by 3 decades, showing that the nonradiative recombination channels can be saturated under strong optical excitation, thus improving the overall emission efficiency. At the maximum power the normalized intensity presents a plateau up to T=150K, that is usually interpreted as a 100% IQE regime. Beyond T=150K additional nonradiative processes become fast enough to overcome the radiative recombination probability: it is understood as a thermally-activated diffusion of the QW excitons towards nonradiative defects. This effect affects the emission efficiency in a similar way for all excitation powers. At low excitation power the slight increase of the normalized intensity below 100K is attributed to an improved efficiency of the QW exciton formation through phonon emission.

It is interesting to evaluate the IQE under the same excitation conditions as in the microlaser operation. This is why we have also measured the ratio $I(300K)/I(5K)$ of the 20 QW active layer under the 400-ps pulsed laser excitation at 4.66eV/266 nm. Even if the excitation conditions and the number of QWs is different, this measurement is compared to the CW excitation at 5.07eV/244 nm on figure S2.d.

We conclude from these measurements that the IQE is not an intrinsic feature of the active layer, and it strongly depends on the excitation conditions (laser energy, power density, …). Assuming that the nonradiative processes are negligible at T=5K and the highest excitation power density, the

measured ratio $I(300K)/I(5K)$ is probably an optimistic estimate of the IQE of the active layer, ranging between 10% at low power and 40% at the highest accessible cw power ($P = 6.6\ 10^4\ W.cm^{-2}$). The same estimate of the IQE reaches 80% under the pulsed excitation identical to the microdisk laser operation. In a recent work on AlGaN/AlN QW ridge lasers[6] an IQE of 10% was obtained in the linear regime, also measured under strong excitation power density. This value appeared as the minimum IQE to reach laser action in the device; it required a drastic improvement of the dislocation density, down to $5.10^8\ cm^{-2}$, i.e. 100 times lower than in our case.

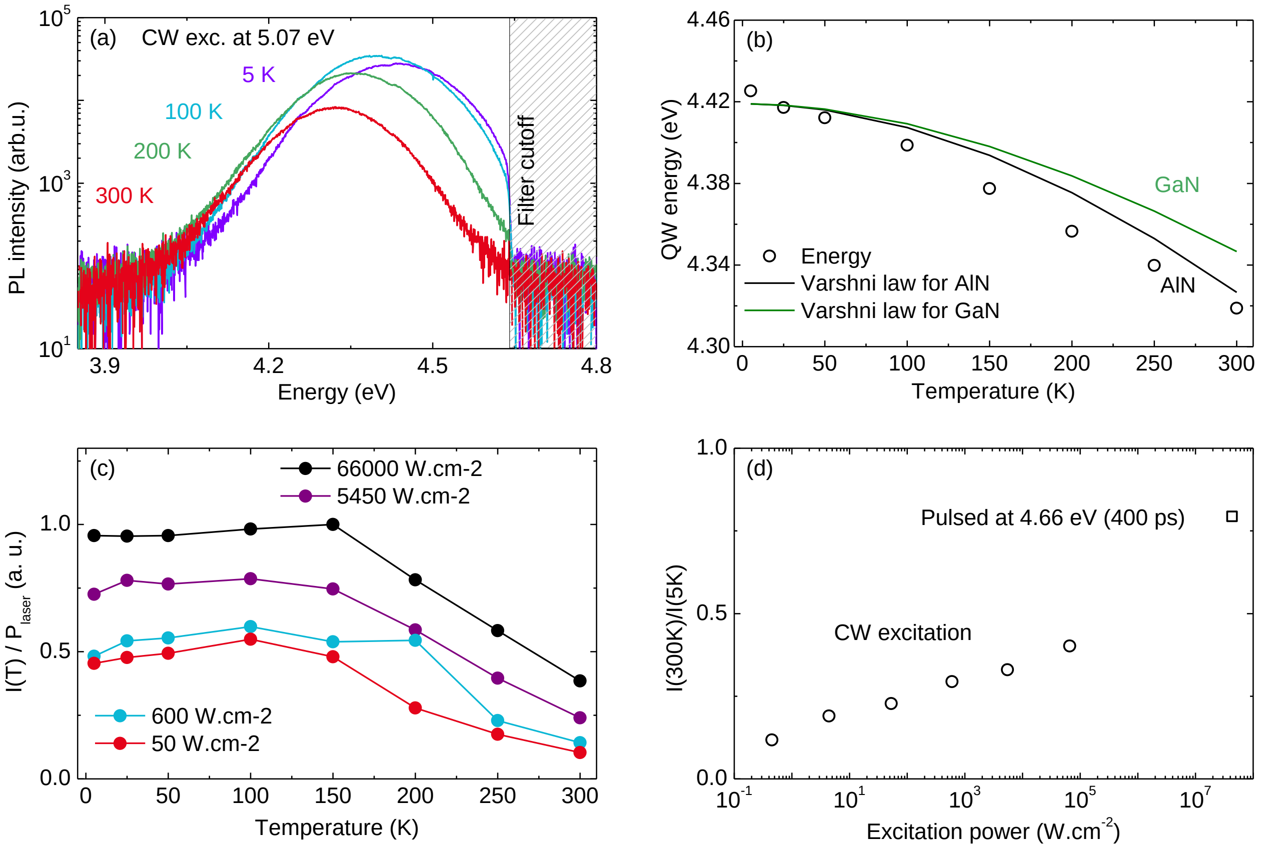


**Figure S2: Temperature dependence of the emission of a single 2.8 ML QW**

(a) PL spectra of a single GaN/AlN QW vs temperature; (b) QW emission energy (dots) vs. temperature, compared to the AlN and GaN Varshni laws (plain lines); (c) QW emission intensity vs. temperature for increasing excitation power density under identical excitation and collection configurations; (d) Ratio of the QW emission intensity at T=300K and 5K under CW macro-PL, CW µPL (circles), and 400 ps-pulses µPL (square) excitation conditions.

## References


1. Li, X.-H. *et al.* Low-threshold stimulated emission at 249 nm and 256 nm from AlGaN-based multiple-quantum-well lasers grown on sapphire substrates. *Appl. Phys. Lett.* **105,** 141106 (2014).
2. Jmerik, V. N. *et al.* Low-threshold 303 nm lasing in AlGaN-based multiple-quantum well structures with an asymmetric waveguide grown by plasma-assisted molecular beam epitaxy on c-sapphire. *Appl. Phys. Lett.* **96,** 141112 (2010).
3. Wunderer, T. *et al.* Pseudomorphically Grown Ultraviolet C Photopumped Lasers on Bulk AlN Substrates. *Appl. Phys. Express* **4,** 092101 (2011).
4. Tamboli, A. *et al.* Room-temperature continuous-wave lasing in GaN/InGaN microdisk. *Nat. Photon* **1**, 61-64 (2007)
5. Simeonov, D. *et al.* Blue lasing at room temperature in high quality factor GaN/AlInN microdisks with InGaN quantum wells. *Appl Phys Lett* **90,** 061106–3 (2007).
6. Martens, M. *et al.* Performance Characteristics of UV-C AlGaN-Based Lasers Grown on Sapphire and Bulk AlN Substrates. *IEEE Photonics Technol. Lett.* **26,** 342–345 (2014).
7. Rosencher, E. & Vinter B., Optoelectronics, Cambridge University Press (2002)
8. Grandjean, N. *et al.* Built-in electric-field effects in wurtzite AlGaN/GaN quantum wells. *J. Appl. Phys.* **86,** 3714–3720 (1999).
9. Kalliakos, S., Lefebvre, P. & Taliercio, T. Nonlinear behavior of photoabsorption in hexagonal nitride quantum wells due to free carrier screening of the internal fields. *Phys Rev B* **67,** 205307 (2003).
10. Bürger, M. *et al.* Lasing properties of non-polar GaN quantum dots in cubic aluminum nitride microdisk cavities. *Appl. Phys. Lett.* **103,** 021107 (2013).
11. McCall, S. L., Levi, A. F. J., Slusher, R. E., Pearton, S. J. & Logan, R. A. Whispering-gallery mode microdisk lasers. *Appl. Phys. Lett.* **60,** 289–291 (1992).
12. Srinivasan, K. *et al.* Optical loss and lasing characteristics of high-quality-factor AlGaAs microdisk resonators with embedded quantum dots. *Appl. Phys. Lett.* **86,** 151106 (2005).
13. Woolf, A. *et al.* Distinctive signature of indium gallium nitride quantum dot lasing in microdisk cavities. *Proc. Natl. Acad. Sci.* (2014). doi:10.1073/pnas.1415464111
